# Do younger Sleeping Beauties prefer a technological prince?

**DOI:** 10.1007/s11192-017-2603-8

**Published:** 2017-12-05

**Authors:** Anthony F. J. van Raan, Jos J. Winnink

**Affiliations:** Centre for Science and Technology Studies, Leiden University, Kolffpad 1, P.O. Box 905, 2300 AX Leiden, The Netherlands

**Keywords:** Sleeping Beauties, Patent citations, Time lag, Inventor-author relations, Technological impact, Technological awakening, Scientific awakening

## Abstract

In this paper we investigate recent Sleeping Beauties cited in patents (SB-SNPRs). We find that the increasing trend of the relative number of SBs stopped around 1998. Moreover, we find that the time lag between the publication year of the SB-SNPRs and their first citation in a patent is becoming shorter in recent years. Our observations also suggest that, on average, in the more recent years SBs are awakened increasingly earlier by a ‘technological prince’ rather than by a ‘scientific prince’. These observations suggest that SBs with technological importance are ‘discovered’ earlier in an application-oriented context. Then, because of this earlier recognized technological relevance, papers may be cited also earlier in a scientific context. Thus early recognized technological relevance may ‘prevent’ papers to become an SB. The scientific impact of Sleeping Beauties is generally not necessarily related to the technological importance of the SBs, as far as measured with number and impact of the citing patents. The analysis of the occurrence of inventor-author relations as well as the citation years of inventor-author patents suggest that the scientific awakening of Sleeping Beauties only rarely occurs by inventor-author self-citation.

## Introduction

A ‘Sleeping Beauty in Science’ is a publication that goes unnoticed (‘sleeps’) for a long time and then, almost suddenly, attracts a lot of attention (‘is awakened by a prince’). This phenomenon of ‘delayed recognition’ attracted the attention of Eugene Garfield already more than 45 year ago, in the early history of the Science Citation Index. With an essay ‘Would Mendel’s work have been ignored if the Science Citation Index was available 100 years ago?’ Garfield opened in 1970 the debate on unnoticed scientific breakthroughs and therefore uncited by contemporary colleague-researchers (Garfield 1970, 1980, 1989, 1990). Particularly, delayed recognition was linked to ‘premature discovery’ or ‘being ahead of time’, i.e., publishing work that is too far outside the body of knowledge at the time of publication (Stent 1972; Garfield 1980). We refer to our earlier paper (van Raan 2015) for a comprehensive overview of the literature on Sleeping Beauties (SBs). In that paper we discussed the results of an extensive analysis of Sleeping Beauties in physics, chemistry, and engineering and computer science (referred to as the three main fields) in order to find out the extent to which Sleeping Beauties are application-oriented and thus are potential Sleeping Innovations. We found that more than half of the SBs are application-oriented.

In a next paper (van Raan 2017a) we took a further step by investigating whether the Sleeping Beauties in physics, chemistry, and engineering and computer science are also cited in patents, i.e., SBs as scientific non-patent references (SNPR) in patents. One of our central topics was the time lag between the publication year of the SB-SNPRs and their first citation in a patent. We found evidences that this time lag was becoming shorter in recent years. In this paper we investigate this further by using as recent as possible SBs cited in patents. Furthermore, we focus on an interesting phenomenon: who will be first, the scientific or the technological prince?

The structure of this paper is as follows. We first discuss the selection of specific sets of SBs, the data collection and numbers of identified SBs as a function of time. Next we discuss the matching of the SBs with patent citation data in order to find SBs cited in patents and to analyze the time lag between publication year of the SB and the year of the first patent citation. We also highlight the technological impact of the patents that cite the SBs. Finally, we summarize our findings and discuss further research.

## Recent trends in the occurrence of Sleeping Beauties

### Choice of sets of Sleeping Beauties

In the foregoing papers (van Raan 2015, 2017a, b) we discussed our fast and efficient search algorithm written in SQL which can be applied to the CWTS enhanced Web of Science (WoS) database. With this algorithm we can tune the following four main variables: (1) *length of the sleep* in years after publication (***s***); (2) *depth of sleep* in terms of a maximum citation rate during the sleeping period (***cs***_max_); (3) *awake* period in years after the sleeping period (***a***_min_ and ***a***_max_); and (4) *awake intensity* in terms of a minimum citation rate during the awake period (***ca***_min_). We define ***cs***_max_ = 0 as a coma, ***cs***_max_ = 0.5 as a very deep sleep, and ***cs***_max_ = 1.0 as a deep sleep.

For a proper analysis of the SBs, we need a total time period equal to sleeping period plus awakening period. Clearly, the longer ***s***, the less publication years we have for our investigation. For instance, if ***s*** = 20, we need a time period 20 + 5 = 25 years. Given that our database is updated up till 2016, and that we want to focus our analyses on an as recent as possible time period (say, from 1990), only publication years (publ y) 1990, 1991, and 1992 can be used.

We identified Sleeping Beauties in the fields of physics, chemistry, engineering and computer science (WoS fields are given in Table 3 of the "Appendix") with the following parameters:

- ***s*** = 20; ***cs***_max_ = 0.0, 0.5, and 1.0; ***a***_min_ = ***a***_max_ = 5; ***ca***_min_ = 5.0; publ y: 1990–1992;
- ***s*** = 15; ***cs***_max_ = 0.0, 0.5, and 1.0; ***a***_min_ = ***a***_max_ = 5; ***ca***_min_ = 5.0; publ y: 1990–1997;
- ***s*** = 10; ***cs***_max_ = 0.0, 0.5, and 1.0; ***a***_min_ = ***a***_max_ = 5; ***ca***_min_ = 5.0; publ y: 1990–2002;
- ***s*** = 5; ***cs***_max_ = 0.0, 0.5, and 1.0; ***a***_min_ = ***a***_max_ = 5; ***ca***_min_ = 5.0; publ y: 1990–2007.

Having identified with help of our SQL search algorithm the SBs that meet the above parameters, we are able to determine the annual numbers of these SBs. We discuss these quantitative aspects in detail in the next section.

### Numbers and trends

The total number of publications covered by the WoS increases constantly over the measuring period. Obviously, if more papers in a given field are published, the number of SBs will, in principle, also increase. If however the number of SBs would increase less than the total number of publications in the given fields, then this could be an indication that the probability for a publication to become a SB decreases. But, of course, this remains to be seen. To examine the above considerations more closely, we first counted the annual number of publications for the period 1990–2007 in physics, chemistry, engineering and computer science. The results are given in Table 1. We used 1990 as the index year.

**Table 1:** Number of WoS-covered publications in physics, chemistry, engineering and computer science

Publ y	Number	Factor
1990	287,117	1.00
1991	307,008	1.07
1992	311,216	1.08
1993	327,490	1.14
1994	355,411	1.24
1995	374,021	1.30
1996	392,728	1.37
1997	403,665	1.41
1998	408,521	1.42
1999	413,328	1.44
2000	415,778	1.45
2001	422,054	1.47
2002	434,495	1.51
2003	462,289	1.61
2004	493,884	1.72
2005	520,804	1.81
2006	540,524	1.88
2007	545,268	1.90
2008	564,709	1.97

**Fig. 1:**
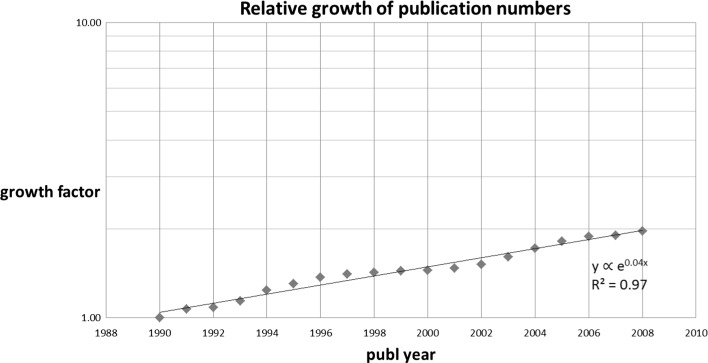
Trend of the number of WoS-covered publications in physics, chemistry, engineering and computer science (index: 1990)

In Fig. 1 we show this annual trend (again 1990 as index year). Clearly, there is an exponential growth of the physics, chemistry, engineering and computer science literature covered by the WoS of about 4% per year.

The annual numbers of SBs are determined with our SQL search algorithm. On the basis of the growth factors given in Table 1 we normalized these annual numbers of the identified SBs, see Table 2 where the absolute (real) and the relative (normalized) numbers are given.

**Table 2:** Absolute (real) and the relative (normalized) numbers of the identified SBs

	*cs*(max)					
	0.0		0.5		1.0	
	Abs	Rel	Abs	Rel	Abs	Rel
*s* = *20*						
1990	0	0	10	10	40	40
1991	0	0	11	10	50	47
1992	0	0	11	10	65	60
*s* = *15*						
1990	0	0	10	10	40	40
1991	0	0	9	8	44	41
1992	0	0	13	12	56	52
1993	0	0	9	8	52	46
1994	0	0	12	10	74	60
1995	1	1	14	11	106	81
1996	1	1	16	12	103	75
1997	1	1	12	9	111	79
*s* = *10*						
1990	0	0	7	7	39	39
1991	0	0	6	6	36	34
1992	0	0	15	14	51	47
1993	0	0	14	12	66	58
1994	1	1	19	15	72	58
1995	0	0	19	15	98	75
1996	1	1	21	15	138	101
1997	1	1	27	19	160	114
1998	1	1	25	18	136	96
1999	2	1	30	21	154	107
2000	1	1	41	28	206	142
2001	1	1	29	20	164	112
2002	1	1	29	19	183	121
*s* = *5*						
1990	1	1	28	28	177	177
1991	6	6	35	33	183	171
1992	7	6	29	27	179	165
1993	2	2	30	26	143	125
1994	2	2	28	23	167	135
1995	3	2	37	28	228	175
1996	10	7	75	55	313	229
1997	9	6	78	55	401	285
1998	19	13	132	93	581	408
1999	9	6	131	91	659	458
2000	7	5	114	79	700	483
2001	9	6	123	84	683	465
2002	15	10	132	87	723	478
2003	12	7	129	80	712	442
2004	18	10	128	74	788	458
2005	14	8	136	75	829	457
2006	23	12	145	77	879	467
2007	10	5	115	61	782	412

**Fig. 2:**
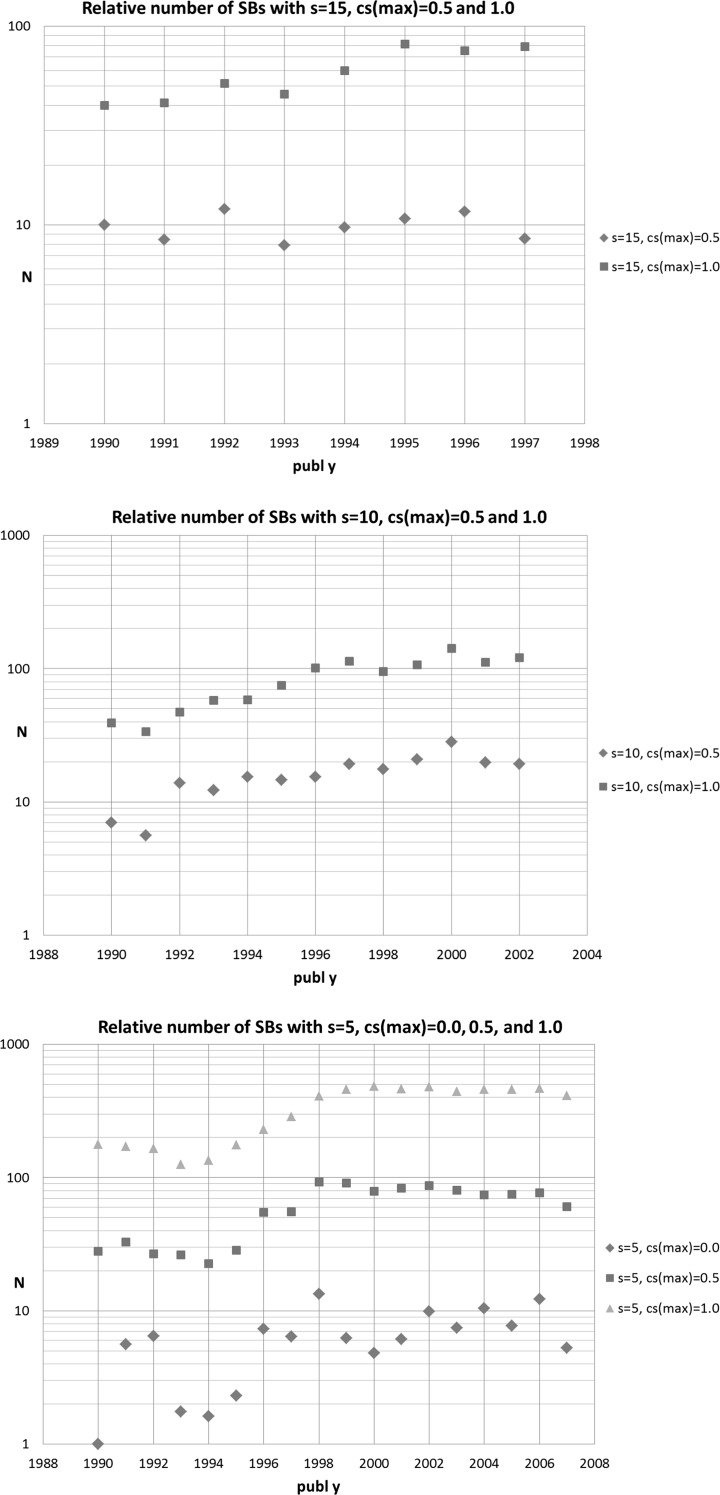
Trend in the relative (normalized) number of SBs. Upper part: SBs with ***s*** = 15, ***cs***_max_ = 0.5 and 1.0; middle part: SBs with ***s*** = 10, ***cs***_max_ = 0.5 and 1.0; lower part: SBs with ***s*** = 5, ***cs***_max_ = 0.0, 0.5 and 1.0

In Fig. 2 we show the relative annual trends. Given the very low numbers in the case of the very long sleeping period ***s*** = 20, and in addition the short measuring period for this sleeping period, we present the trends for ***s*** = 15, 10, and 5, and ***cs***_max_ = 0.5 and 1.0, and in the case of ***s*** = 5 also for ***cs***_max_ = 0.0.

SBs with ***s*** = 5 have the shortest sleeping period and thus they can be analyzed for the most recent times, until 2007. Our results show that from around 1998 the relative number of SBs is not increasing anymore. This is an interesting and perhaps also counterintuitive finding: it appears that, probably, the expanding worldwide facilities to access scientific publications stopped the earlier increasing trend. But nevertheless it apparently does not prevent that a more or less constant fraction of publications still becomes an SB.

## Scientific or technological awakening?

### Time lags between publication year and year of first patent citation

Patents are documents with a legal status to describe and claim technological inventions in which, similar to scientific publications, references are given. These references concern mainly earlier patents (‘prior art’) in order to prove novelty in view of the existing technological developments. Generally, but to a lesser extent, patents also have references to non-patent items, particularly scientific publications, the ‘scientific non-patent references’ (SNPRs). References in scientific publications are the sole responsibility of the authors. References in patents, however, come from two sources: they can be given by both the inventors as well as by the patent examiners. In this study patent citations of both sources are considered to have the same function of linking science with technology. Clearly, these SNPRs represent a bridge between science and technology although they do not necessarily indicate the direct scientific basis of the invention described in the patent. Nevertheless, many studies (for an overview see for instance Callaert et al. 2014) emphasize the importance of further research of the role of SNPRs in relation to the patented technological invention. In this study we elaborate further on our previous work on SBs that are SNPRs (SB-SNPRs) by focusing on as recent as possible SBs.

Patent data were collected by searching the EPO Worldwide Patent Statistical Database (PATSTAT), Spring 2016 version. We group patents describing the same invention in ‘patent families’^1^ to prevent double counting. In order to find out whether an SB is cited by patents, we matched all SBs on the basis of their WoS UT-codes with the citations given in patents. For more details we refer to Winnink and Tijssen (2014). We choose two sets for matching with patent data: (1) SBs sleeping long (***s*** = 10) and deep (***cs***_max_ = 0.5), and (2) SBs sleeping short (***s*** = 5) but in coma (***cs***_max_ = 0.0). We find, as follows from Table 1, 282 SBs for set 1, and 176 SBs for set 2. As we discussed earlier, in both cases ***a***_min_ = ***a***_max_ = 5 and ***ca***_min_ = 5.0.

With the matching algorithm (written as an SQL-query applicable to the PATSTAT database) we find that 44 of the 282 SBs in set 1 are cited by patents, i.e., 16% (2 of these SB-SNPRs are a note, and one is a review paper); and that 18 of the 176 SBs in set 2 are cited by patents, i.e., 10% (none of these SB-SNPRs are notes or reviews). Similar to citations given by publications, also the number of citations by patents is characterized by a skew distribution. For instance, about half of the SB-SNPRS in set 1 are cited by 1 or 2 patents, and five (of the 44) are cited by 5 of more patents. In total, the 44 SB-SNPRs in set 1 are cited by 119 patents. In Set 2 the large majority of SB-SNPRs are cited by only 1 patent and one is cited by 17 patents. In total, the 18 SB-SNPRs in set 2 are cited by 37 patents.

**Fig. 3:**
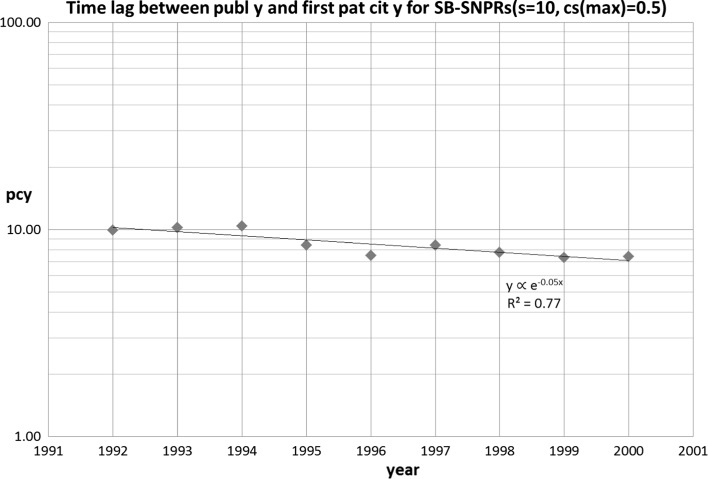
Time lag between publication year and first patent citation year (**pcy**) for the set 1 SB-SNPRs (***s*** = 10, ***cs***_max_ = 0.5). The years indicated on the abscissa are the middle years of the successive 5-years periods (see main text)

**Fig. 4:**
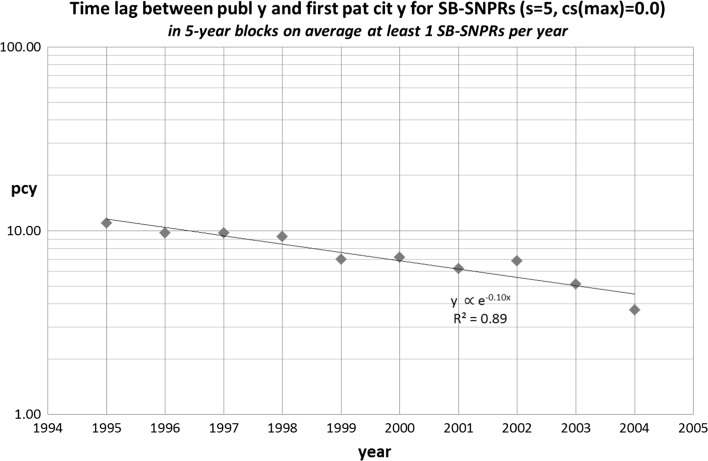
Time lag between publication year and first patent citation year (**pcy**) for the set 2 SB-SNPRs (***s*** = 5, ***cs***_max_ = 0.0). The years indicated on the abscissa are the middle years of the successive 5-years periods (see main text)

In a next step we determined for the identified SB-SNPRs the filing year of the citing patents. The difference between the filing year of the patent that is the first citer of the SB-SNPR and the publication year of the SB-SNPR defines the time lag between publication and the first citation by a patent (***pcy***). This time lag ranges in set 1 from 1 to 18 years (average 8.5, SD = 4.3), and in set 2 from 1 to 14 years (average 6.7, SD = 4.4). The average values of ***pcy*** relates to a long measuring period. In order to find out if there is a trend in the course of time, we calculated averages of ***pcy*** for successive, partly overlapping 5-year periods. In the case of the long sleeping SBs (***s*** = 10) these periods are 1990–1994, 1991–1995,…1998–2002. In Figs. 3 and 4 we present the results for set 1 and set 2, respectively. Remarkably, for the set 1 SB-SNPRs the time lag (***pcy***) becomes rapidly shorter in the measured period (1990–2002) than the sleeping time (***s*** = 10). For the set 2 SB-SNPRs we see that initially the time lag is considerably higher (around 10 years) than the sleeping period (***s*** = 5). But even more rapidly than in the case of set 1, the time lag decreases, and also for this set 2 the time lag becomes shorter than the sleeping period (in this case 5 years). Both observations suggest that, on average, in the more recent years SBs are awakened more and more earlier by a ‘technological prince’ rather than by a ‘scientific prince’.

On the basis of the scope of the journals in which the SBs are published we find that 174 of the 282 SBs (62%) in set 1 are typically application-oriented, for the 44 SB-SNPRs in this set this percentage is considerably higher (91%). In set 2 these percentages are 66 and 78%, respectively, so comparable with the results for set 1, but less pronounced.

### Are Sleeping Beauties cited by high impact patents?

Just as in the case of publications, also patents show a wide variety of impact. Only a relatively small amount of patents represents important technological breakthroughs (Albert et al. 1991). Patent-to-patent citations provide a first indication of the importance of the cited patents (Trajtenberg 1990), particularly if they are highly cited and belong to, for instance, the top-10% cited patents in their field. Harhoff et al. (1999) found that patents renewed to full-term (which is the maximum duration of the patent protection, mostly 20 years) were significantly more highly cited than patents allowed to expire before their full term. The higher an invention’s economic value estimate was, the more the patent was subsequently cited. For an overview of patent citation analysis studies we refer to van Raan (2017b).

In this section we analyze the extent to which the patents that cited our SB-SNPRs are themselves cited by other patents. In the foregoing section we discussed that the 44 SB-SNPRs in set 1 (sleep is long (***s*** = 10) and very deep (***cs***_max_ = 0.5)) have been cited by in total 119 patents of which the majority is cited by other patents. These cited patents relate to 39 SB-SNPRs. Thus the majority (82%) of the SB-SNPRs in set 1 do have patents that are cited by other patents after their publication within a 5-year citation window (longer citation windows such as 10 or 15 years can only be applied to the oldest SB-SNPRS in our set). In total, the patents that cite the SB-SNPRs are themselves cited 1530 times by other patents within the 5 years window. Taking the total of patent citations for each SB-SNPR we find that the distribution is very skew. For two SB-SNPRs their citing patents are cited 633 and 126 times respectively, for five SB-SNPRs it is between 50 and 100 times, for 18 SB-SNPRS between 10 and 50, and the 14 remaining SB-SNPRs it is between 1 and 10.

**Fig. 5:**
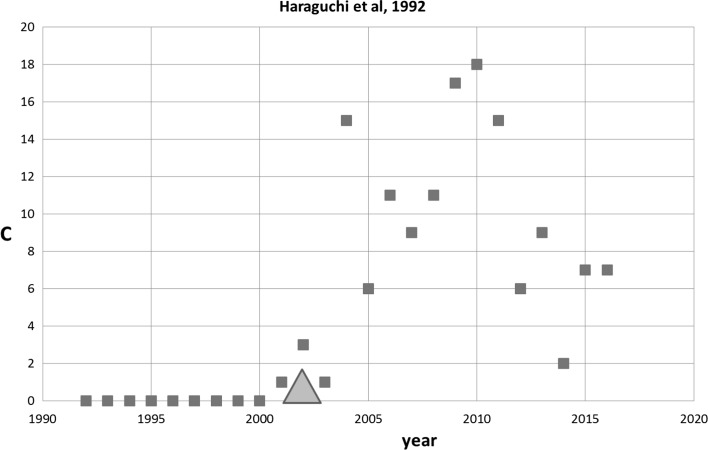
Haraguchi SB-SNPR published in 1992 (very deep sleep ***cs*** = 0.1). Red squares indicate number of citations. The green triangle indicates the year of first citation in a patent (2002). (Color figure online)

The above mentioned SB-SNPR with 633 patent citations is published in 1992 and it describes a new technique with which for the first time p–n-junctions were formed in GaAs quantum wire crystals (Haraguchi et al. 1992). The high-impact patent cited this SB-SNPR 10 years after its publication, so the technological and scientific awakening happened around at the same time. Figure 5 shows the citation history of the Haraguchi SB-SNPR. After a long (***s*** = 10) and very deep sleep (***cs***_max_ = 0.5) the scientific awakening is just before the first patent citation in 2002. After an increase in citations by other publications until 2010, the scientific impact of the Haraguchi SB-SNPR decreases rapidly. The technological impact of its citing patents, however, continues to increase substantially from 633 patent citations in the 5 years window to 1215 in a 15 years window (covering the time period up to and including 2016).

**Fig. 6:**
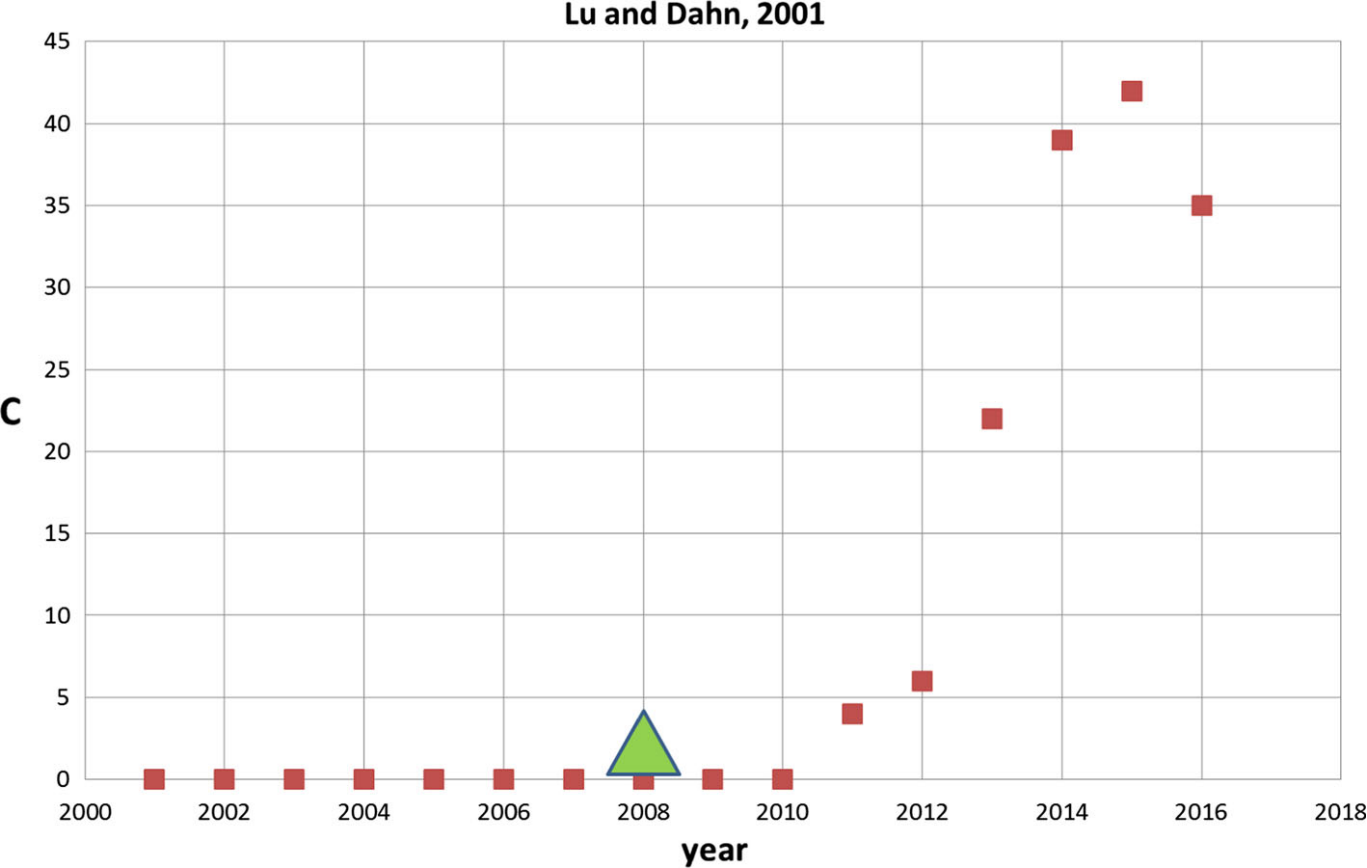
Lu and Dahn SB-SNPR published in 2001 (coma sleep ***cs*** = 0.0). Red squares indicate number of citations. The green triangle indicates the year of first citation in a patent (2002). (Color figure online)

Another interesting and more recent case in set 1 is the SB-SNPR by Lu and Dahn (2001). It is not cited at all during its sleep period of 10 years, and thus this SB-SNPR is in fact characterized by a very long ‘coma’. The authors discuss an X-ray diffraction study to find the molecular structure of a metal oxide that is crucial in the construction of high performance cathodes for rechargeable sodium ion batteries. During this coma period the first patent citation is received 7 years after publication. Figure 6 shows the citation history of the Lu and Dahn SB-SNPR. We see that the first citing patent act as the technological prince, and this happens before the scientific prince arrives. After the scientific awakening the impact of this SB-SNPR in terms of citations by other papers increases very rapidly. The impact of the citing patents, however, is low as compared to the Haraguchi case (17 patent citations in the 5 year window) and increases hardly anymore. Thus, the Lu and Dahn and the Haraguchi SB-SNPRs represent two quite opposite cases.

For SB-SNPRs in set 1 we found no significant correlation between the number of citations by other publications during the awake period and both the number of patents that cite the SB-SNPR as well as the number of time these patents are cited themselves by other patents. This means that the scientific impact of Sleeping Beauties is generally not related to the technological importance of the SBs, as far as measured with number and impact of the citing patents.

For set 2 [sleep is short (***s*** = 5) but coma (***cs***_max_ = 0.0)] the 18 SB-SNPRs have been cited by in total 37 patents. The majority of the 18 SB-SNPRs are cited by 1 to 3 patents and one is cited by 17 patents of which the majority is cited by other patents. These cited patents relate to 15 SB-SNPRs. Thus, also in set 2 the majority (83%) of the SB-SNPRs do have patents that are cited by other patents after their publication. Similar to our analysis of set 1, we apply also here a 5-year citation window. Taking the total of patent citations for each SB-SNPR we again find that the distribution is very skew. For one SB-SNPR its citing patents are cited 351 times, for four other SB-SNPRs it is between 10 and 50 times, and the for the remaining SB-SNPRs it is between 1 and 6. The SB-SNPR with 351 patent citations is published in 1991 and concerns a novel type switch chip with algorithms implemented in hardware (Katevenis et al. 1991). Also this technologically important SB-SNPR is not a paper with a very high scientific impact in terms of citations by other publications, but still it belongs to the top-25% of set 2. Scientifically this SB-SNPR slept in coma for 5 years, but she awakened technologically already 2 years after her publication being cited by one of the high impact patents.

**Fig. 7:**
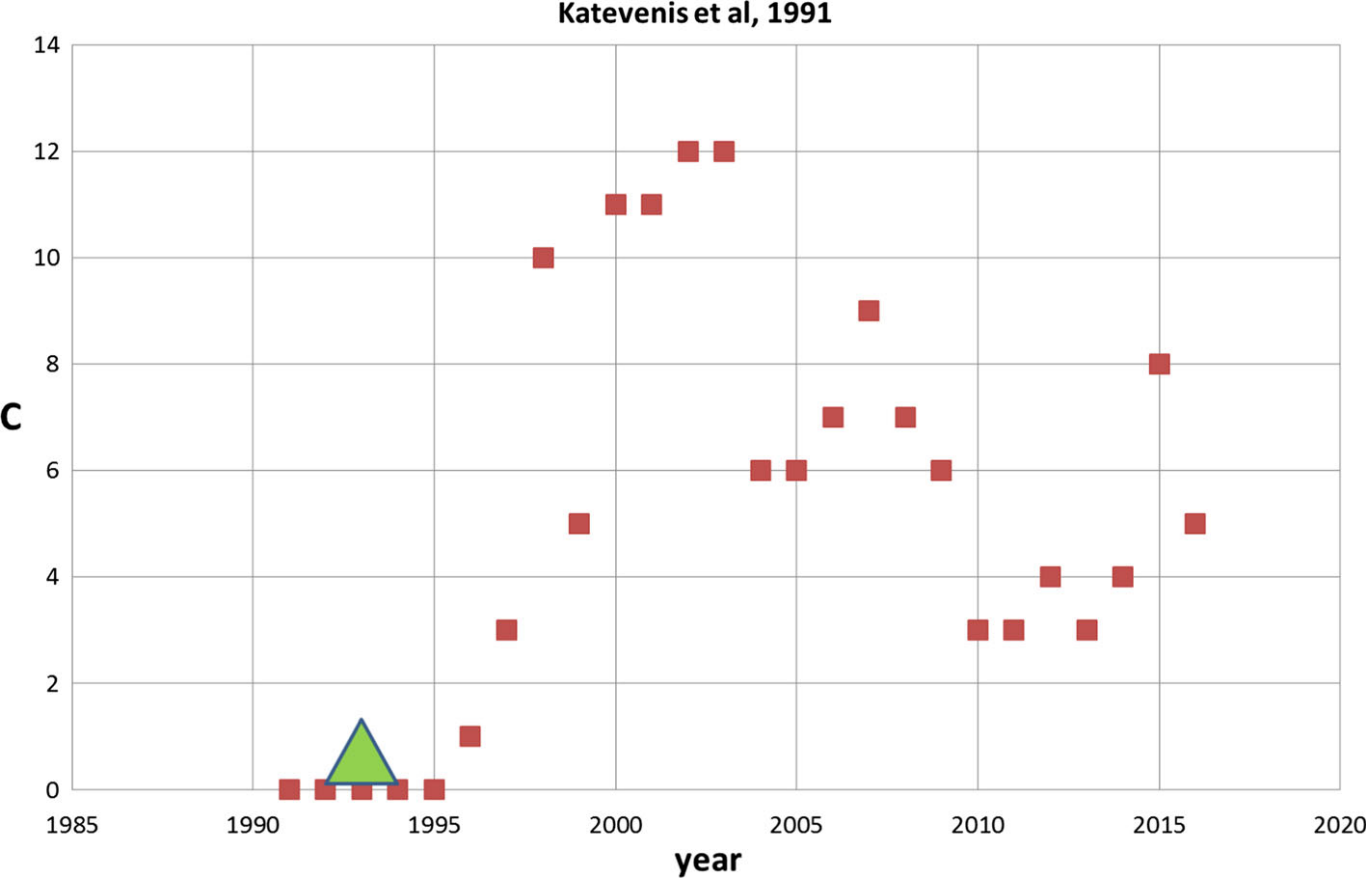
Katevenis SB-SNPR published in 1991 (coma ***cs*** = 0.0). Red squares indicate number of citations. The green triangle indicates the year of first citation in a patent (1993). (Color figure online)

Figure 7 shows the citation history of this Katevenis SB-SNPR. After a short (***s*** = 5) coma sleep (***cs*** = 0.0) the scientific awakening is 3 years after the technological awakening, i.e., the first patent citation in 1993. After a rapid increase in citations by other publications until 2003, the scientific impact of the Katevenis SB-SNPR decreases. The technological impact of its citing patents, however, increases from 351 patent citations in the 5 years window to 585 in a 15 years window (covering the time period up to and including 2016).

**Fig. 8:**
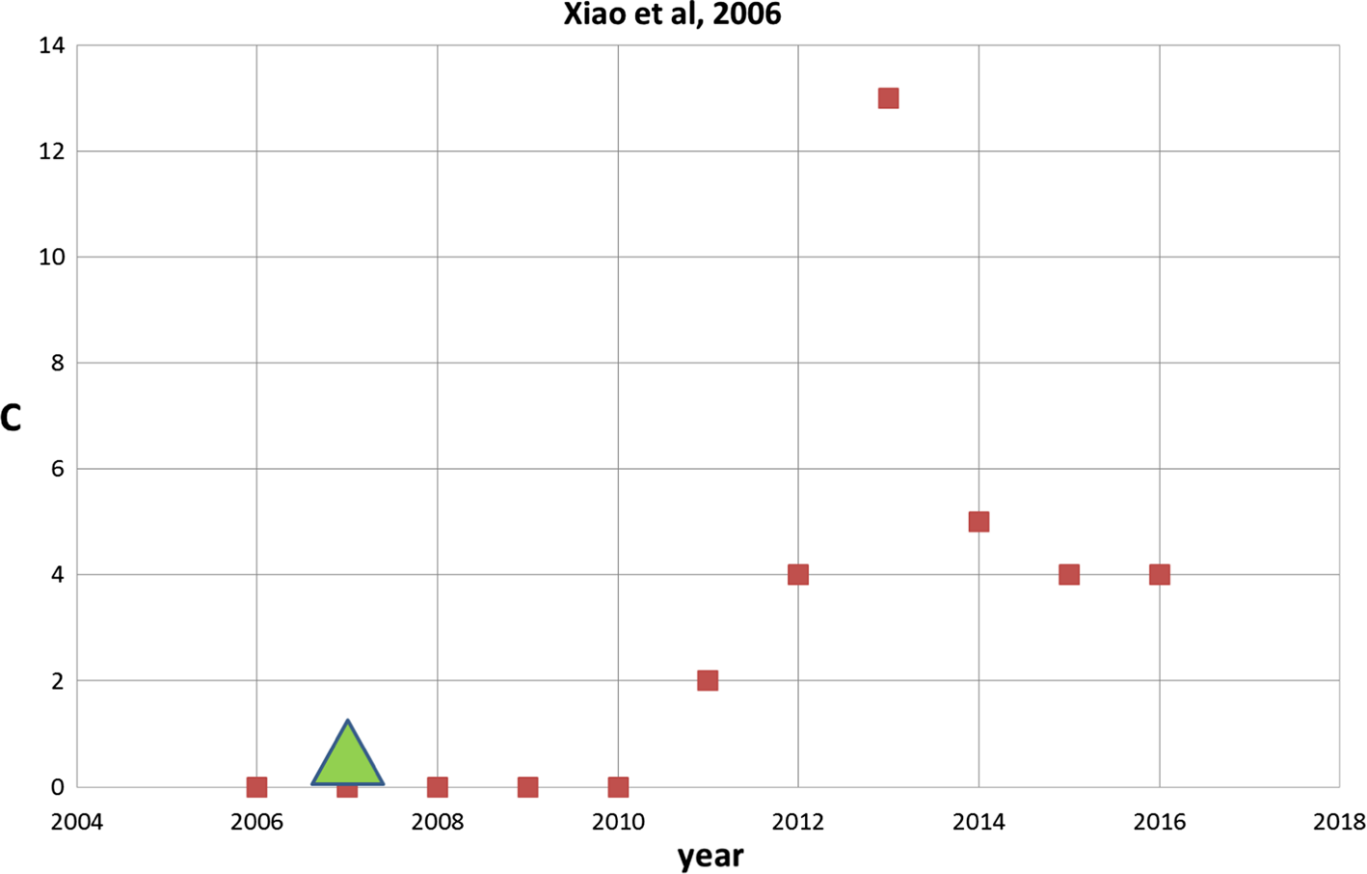
Xiao SB-SNPR published in 2006 (coma ***cs*** = 0.0). Red squares indicate number of citations. The green triangle indicates the year of first citation in a patent (2007). (Color figure online)

One of the most recent SB-SNPRs in set 2 is the paper by Xiao et al. (2006). The authors discuss a method on the optical flow estimation related to the motion of objects in computerized vision such as video and TV. Figure 8 shows the citation history of the Xiao SB-SNPR. We see that already a year after its publication the first (and only) citing patent was received. Also in this case clearly a technological prince, arrives before the scientific awakening. After the scientific awakening the impact of the Xiao SB-SNPR in terms of citations by other papers increases during a few years and then decreases. The impact of the citing patent, however, is still increasing (29 citations by other patents in the 5-years window, and 61 in a 10-years window) showing the importance of the technology related to the Xiao SB-SNPR.

### Inventor-author relations

In the foregoing study (van Raan 2017a) we investigated the extent to which SB-SNPRs are cited in patents of which at least one of the inventors is also an author of the cited SB-SNPR. Such an inventor-author self-citation may trigger a ‘self-awakening’ of the Sleeping Beauty. We concluded however that only for a small minority (5%) of the Sleeping Beauties that are cited in patents the authors are also inventors of the technology described in the citing patent.

This study confirms the earlier observations. We find that in set 1 (long (***s*** = 10) and very deep (***cs***_max_ = 0.5) sleep), the 44 SB-SNPRs with in total 141 authors are cited by 117 patents which have in total 425 inventors. Only in ten cases these inventors are also author, and this concerns five different SB-SNPRs (of the 44). On the basis of the patent citation year of the inventor-author patent we find that for four of the five SB-SNPRs the inventor-author patent did not trigger the scientific wakening. For one SB-SNPR the inventor-author patent citation took place in the year of the scientific awakening, so here the inventor-author patent may have triggered the scientific awakening. Thus based on our observations in set 1 we first conclude that inventor-author self-citation is quite rare, and, secondly, that in most of the cases where inventor-author self-citation occurred, we found no trigger effect for the scientific awakening.

In set 2 (short sleep (***s*** = 5) but in coma (***cs***_max_ = 0.0)) the 18 SB-SNPRs with in total 57 authors are cited by 37 patents which have in total 109 inventors. Only three of these inventors are also author, and this concerns three different SB-SNPRs. One of these three is the Katevenis SB-SNPR. Katevenis is the inventor of a patent that cites the Katevenis Sleeping Beauty in 1995 (Katevenis et al. 1991), 4 years after the publication of the SB. However, this inventor-author self-citation is not the first patent citation of the Katevenis SB; as discussed in the foregoing section and shown in Fig. 7, the Katevenis SB was first cited by a patent (of which Katevenis was not an inventor) 2 year after its publication. This first patent citation clearly did not trigger the scientific awakening. Remarkably, the inventor-author self-citation by the Katevenis patent is in the year just before the scientific awakening. For the two other SB-SNPRs the inventor-author self-citation took place after the scientific awakening.

## Conclusions

We investigated characteristics of Sleeping Beauties that are cited in patents (SB-SNPRs) with a focus on recent cases. In line with earlier observations in our previous study (van Raan 2017a) we find that also in the case of recent Sleeping Beauties patent citation may occur before or after the scientific awakening.

Another observation in the previous study was that the average time lag between the publication year of an SB-SNPR and its first citation in a patent appears to decrease in the 1980s and early 1990s. In this study we find that this trend continues in the more recent years, the later 1990s and the early 2000s. In other words, the time lag between the publication year of the SB-SNPRs and their first citation in a patent is becoming shorter in recent years. This means that, on average, in the more recent years SBs are awakened increasingly earlier by a ‘technological prince’ than by a ‘scientific prince’. Thus, we think that the question posed in the title of this paper can be answered with yes. We discussed examples of this phenomenon, particularly the cases with a high technological impact of the citing patents.

At the same time, we find that the increasing trend of the relative number of SBs stopped around 1998. The above observations suggest the following possible scenario. We think that SBs with technological importance are ‘discovered’ increasingly earlier in an application-oriented context. Then, because of this earlier recognized technological relevance, papers may be cited also earlier in a scientific context. Thus early recognized technological relevance may ‘prevent’ papers to become an SB.

In this study we also find that the scientific impact of Sleeping Beauties is generally not related to the technological importance of the SBs, as far as measured with number and impact of the citing patents. In both sets of Sleeping Beauties analyzed in this study we do find a relation of the scope of the journal in which the SBs are published with patent citations: the SB-SNPRs are significantly more published in application-oriented journals as compared to the SB-nonSPNRs. Given the fact that patents can be considered ‘solutions to technical problems’ this focus of SB-SPNRs on application-oriented journals seems logical.

In our previous study we found that only for a small minority of the Sleeping Beauties that are cited in patents the authors are also inventors of the technology described in the citing patent. This study confirms the earlier observation. In addition, the analysis of the citation years of the inventor-author patents suggest that the scientific awakening of Sleeping Beauties only rarely occurs by inventor-author self-citation.

Follow-up research will focus on two crucial issues. First, further evidence is needed to support the observation made in this study that in the more recent years SBs are awakened more and more earlier by a ‘technological prince’ than by a ‘scientific prince’. Secondly, an analysis of the opinions of the SB authors as well as of the ‘princes’ is necessary to answer the question whether a publication became a SB because it was (to far) ahead of its time.

## Appendix

See Table 3.

**Table 3:** WoS Fields codes and names of physics, chemistry, engineering and computer science

Physics	
WoS field code and name	
1	Acoustics
20	Astronomy and Astrophysics
27	Biophysics
35	Thermodynamics
152	Materials science, Biomaterials
153	Materials science, Characterization and testing
154	Materials science, Coatings and films
155	Materials science, Composites
156	Materials science, Textiles
159	Meteorology and atmospheric sciences
168	Nuclear science and technology
175	Optics
185	Physics, Applied
187	Physics, Fluids and plasmas
188	Physics, Atomic, Molecular and chemical
189	Physics, Multidisciplinary
190	Physics, Condensed matter
192	Physics, Nuclear
193	Physics, Particles and fields
195	Physics, Mathematical

Chemistry	
WoS field code and name	
23	Biochemical research methods
24	Biochemistry and molecular biology
36	Chemistry, Applied
37	Chemistry, Medicinal
38	Chemistry, Multidisciplinary
39	Chemistry, Analytical
40	Chemistry, Inorganic and nuclear
41	Chemistry, Organic
42	Chemistry, Physical
57	Crystallography
63	Geochemistry and geophysics
71	Electrochemistry
198	Polymer science

Engineering and computer science	
WoS field code and name	
6	Engineering, Aerospace
28	Biotechnology and applied microbiology
44	Computer science, Artificial intelligence
46	Computer science, Cybernetics
47	Computer science, Hardware and architecture
48	Computer science, Information systems
49	Communication
50	Computer science, Interdisc applications
51	Computer science, Software engineering
52	Computer science, Theory and methods
54	Construction and building technology
75	Energy and fuels
76	Engineering, Multidisciplinary
77	Engineering, Biomedical
78	Engineering, Environmental
79	Engineering, Chemical
80	Engineering, Industrial
81	Engineering, Manufacturing
82	Engineering, Marine
83	Engineering, Civil
84	Engineering, Ocean
85	Engineering, Petroleum
86	Engineering, Electrical and electronic
87	Engineering, Mechanical
97	Food science and technology
119	Instruments and instrumentation
131	Operations research and management science
145	Medical laboratory technology
147	Metallurgy and metallurgical engineering
168	Nuclear science and technology
173	Remote sensing
186	Imaging science and photographic technology
222	Telecommunications
227	Transportation
237	Mining and mineral processing
242	Transportation science and technology
244	Agricultural engineering
245	Critical care medicine
247	Engineering, Geological
248	Integrative and complementary medicine
251	Robotics
252	Nanoscience and nanotechnology
257	Cell and tissue engineering

## Footnotes

1 In this study a patent family is a set of, in legal terms, equivalent patents that describe one and the same invention. This is for instance the case when the same invention is patented in more than one country.
